# Luteolin-Loaded Elastic Liposomes for Transdermal Delivery to Control Breast Cancer: In Vitro and Ex Vivo Evaluations

**DOI:** 10.3390/ph14111143

**Published:** 2021-11-11

**Authors:** Mohammad A. Altamimi, Afzal Hussain, Mohammad AlRajhi, Sultan Alshehri, Syed Sarim Imam, Wajhul Qamar

**Affiliations:** 1 Department of Pharmaceutics, College of Pharmacy, King Saud University, P.O. Box 2457, Riyadh 11451, Saudi Arabia; 436107433@ksu.edu.sa (M.A.); salshehri1@ksu.edu.sa (S.A.); simam@ksu.edu.sa (S.S.I.); 2 Department of Pharmacology and Toxicology, College of Pharmacy, King Saud University, P.O. Box 2457, Riyadh 11451, Saudi Arabia; wqidris@ksu.edu.sa

**Keywords:** luteolin, elastic liposomes, design expert-based optimization, ex vivo permeation and drug deposition, cytotoxicity against MCF-7

## Abstract

The study aimed to prepare and optimize luteolin (LUT)-loaded transdermal elastic liposomes (LEL1-LEL12), followed by in vitro and ex vivo evaluations of their ability to control breast cancer. Various surfactants (Span 60, Span 80, and Brij 35), and phosphatidyl choline (PC) as a lipid, were used to tailor various formulation as dictated by “Design Expert^®^ software (DOE). These were characterized for size, polydispersity index (PDI), and zeta potential. The optimized formulation (OLEL1) was selected for comparative investigations (in vitro and ex vivo) against lipo (conventional liposomes) and drug suspension (DS). Moreover, the in vitro anticancer activity of OLEL1 was compared against a control using MCF-7 cell lines. Preliminary selection of the suitable PC: surfactant ratio for formulations F1–F9 showed relative advantages of Span 80. DOE suggested two block factorial designs with four center points to identify the design space and significant factors. OLEL1 was the most robust with high functional desirability (0.95), minimum size (202 nm), relatively high drug release, increased drug entrapment (92%), and improved permeation rate (~3270 µg/cm^2^) as compared with liposomes (~1536 µg/cm^2^) over 24 h. OLEL1 exhibited a 6.2- to 2.9-fold increase in permeation rate as compared with DS (drug solution). The permeation flux values of OLEL1, and lipo were found to be 136.3, 64 and 24.3 µg/h/cm^2^, respectively. The drug disposition values were 670 µg, 473 µg and 148 µg, for OLEL1, lipo and DS, respectively. Thus, ex vivo parameters were significantly better for OLEL1 compared with lipo and DS which is attributed to the flexibility and deformability of the optimized formulation. Furthermore, OLEL1 was evaluated for anticancer activity and showed maximized inhibition as compared with DS. Thus, elastic liposomes may be a promising approach for improved transdermal delivery of luteolin, as well as enhancing its therapeutic efficacy in controlling breast cancer.

## 1. Introduction

Cancer is a disease with the highest mortality rate second only to cardiovascular disorders [1,2]. Furthermore, Sung H, et al., published statistics based on GLOBOCAN, showing that nearly 20 million new cancer cases and 10 million deaths occurred worldwide in the year 2020 alone. The projection of newly diagnosed cancer cases worldwide is estimated to be nearly 30 million in 2040 [3]. In 2020, 2.3 million women were diagnosed with breast cancer and 568,000 deaths occurred globally [4].

**Figure 1:**
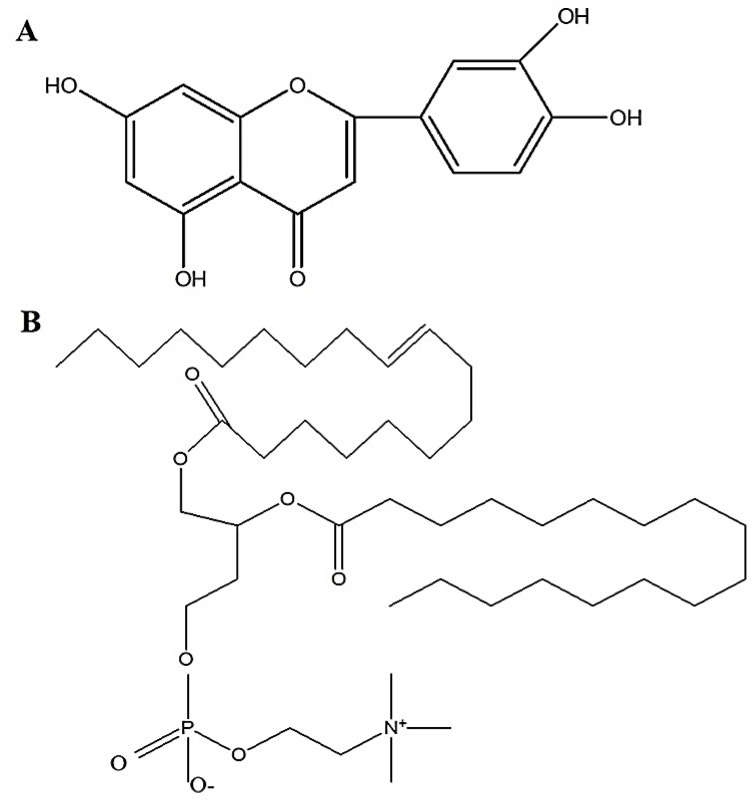
Chemical structures of (**A**) luteolin, and (**B**) phosphatidylcholine of Phospholipon 90G.

Several synthetic and natural drugs have been explored for their therapeutic potential to control breast cancer. However, commercial synthetic or semi-synthetic drugs are associated with various side effects and drug related toxicity. Natural luteolin (LUT) is found in spinach, different peppers, and lettuce. LUT possess anticancer potential despite having various additional therapeutic benefits (antioxidant, anti-inflammatory, antiapoptotic agent). Chemically, the drug is a natural flavone (tetrahydroxy flavone) with four hydroxyl functional groups positioned at 3, 4, 5, and 6 of basic moiety and conjugate acid of 2-(3,4-dihydroxyphenyl)-5-hydroxy-4-oxo-4H-chromen-7-olate luteolin-7-olate(1-) (Figure 1A) [5,6]. Pharmacologically, the drug is a potential antioxidant (free radical scavenger), anti-inflammatory, anti-mutagen, antimicrobial, immunomodulatory, apoptosis inducer, and anti-neoplastic against several cancer types [7,8,9,10]. Pharmaceutically, lipophilic LUT (logP ~ 2.53) is poorly soluble in water (0.0055 mg/mL), unstable in gastric lumen due to acidic environment (pKa ~ 6.5) and is associated with low oral bioavailability (<30%) [11,12]. Considering this context, it is a challenging task to formulate a suitable dosage formulation for oral and parenteral delivery due to poor aqueous solubility in water. Therefore, low molecular weight LUT (286 g/mole) is a suitable drug candidate for transdermal delivery using vesicular nanocarrier to control breast cancer. Transdermal route of administration does overcome barriers as it avoids the first pass metabolism, has direct and local exposure, avoids stability issues related to gastric fluid, and offers improved patient compliance. However, percutaneous drug delivery faces the major challenge of low drug penetration. Topically applied medicines must pass through the stratum corneum (SC) which contain corneocytes in lipid matrix. Thus, the drug must pass through small pore sizes of nearly 30 nm [13]. Liposomes, elastic liposomes, ethosomes, niosomes, and PEGylated liposomes have been explored as several lipophilic compounds for transdermal and topical administration. Abidin et al. investigated enhanced transdermal delivery of LUT via non-ionic niosomes to control arthritis [14]. Similarly, Huang et al. encapsulated luteolin in liposomes and compared the protective effect of liposomes loading LUT, quercetin and kaempferol in term of structure, size, and loading [15]. However, elastic liposomes possess unique benefits over other vesicular systems due to their ultra-deformability, absence of cholesterol, and capability to permeate across microscopic pores of skin for drug access to the dermal region. Physicochemical properties of elastic vesicles depend upon several factors such as (a) the type of surfactant (ionic, non-ionic and amphiphilic), (b) nature of the hydrocarbon chain present in lipid and surfactant (saturated, unsaturated, branching and length), size of surfactant head group (polar, charged or uncharged), concentration, transition temperature of surfactant, and lipophilicity (lipid, surfactant, and drug) [16,17]. Thus, to our knowledge, no report has been published regarding the transdermal delivery of LUT for the treatment of breast cancer.

In this study, we aimed to formulate LUT-loaded elastic liposomes using various surfactants (based on different HLB and transition temperature), optimize them using Design Expert, and evaluate their in vitro parameters. Ex vivo permeation parameters (cumulative permeation rate, enhancement ratio, permeation flux and drug deposition) were investigated using rat skin for comparison against the drug suspension. Finally, the optimized formulation was evaluated for in vitro anticancer activity using MCF-7 cell lines.

## 2. Results and Discussion

### 2.1. Screening of Lipid and Surfactant Ratio

#### 2.1.1. Preliminary Study to Select Lipid and Surfactant Ratio

The basic liposomal formulation contains phospholipid (containing ~ 94% phosphatidylcholine as major constituent as shown in Figure 1B) and surfactant in a specific ratio. Here, formulations were prepared using varied ratios of phosphatidylcholine to surfactants. The selected composition ratios were (PC: Span 60, PC: Span 80 and PC: Brij 35) (Table 1). The basis for selection of the surfactants were hydrophilic lipophilic balance (HLB), which was anticipated to have significant impact on size, % EE, and elastic nature of ELs. Results revealed significant difference in the values of size and PDI when formulated with the selected ratio of PC to specific surfactant based on HLB (low and high) and glass transition temperature (low to high) of surfactant as shown in Table 1. Nonionic Span 80 (HLB ~ 4.3) and Span 60 (HLB ~ 4.7) are expected to impart substantial deformability and flexibility in the lipid bilayer followed by reduced vesicle size and PDI values. Therefore, results showed reduced size of the vesicles (358–170 nm) and PDI (0.62–0.35) values. Also, the change in PDI values may be due to formation of small micelles to some extent [16,18,19]. On the other hand, nonionic (hydrophilic) polyoxyethylene (23) laurylether (Brij 35) with (HLB ~ 16.9) showed an increase in the average vesicle size compared with the lipophilic surfactants with nearly similar PDI values at different ratios [20,21,22]. The disparity in the hydrophilicity between Brij 35 and LUT is attributed to the average larger vesicles size as compared with Span 60 and Span 80. In general, micelles are formed above their CMC value and can coexist with the liposomal formulation leading to reduced size and entrapment efficiency of the formulated vesicles. Therefore, Span 80 was selected as the suitable surfactant for further optimization using the experimental tool (two blocks factorial design with four center points) (Design Expert). In the present study, 30 mg lutein was added to the formulation to get 3 mg per g of total formulation (0.3% *w*/*w*). Moreover, the drug strength per 100 mg of lipid was found to be in the range of 3.2–4.3 mg for the developed formulations (Table 1).

#### 2.1.2. Optimization Using Design Expert

This experimental tool is used for identifying the relationships between independent factors and dependent factors. These processes involve identifying factors, their levels, and their importance. In addition, it can identify the interactions between factors (independent variables) against set responses (dependent variables), which for this study entailed two factors (X_1_ and X_2_) at two levels such as minimum (−1) and maximum (+1). Independent (factors) and dependent variables (responses) were opted considering preliminary results of size and PDI values. Thus, PC (X_1_) and Span 80 (X_2_) were two independent factors against three responses, namely vesicle size (Y_1_), zeta potential (Y_2_), and %EE (Y_3_). Two runs at higher and lower levels were conducted to find the noise in each response and establish signal to noise ratios. This ratio allows for design power estimation where anything above 80% is deemed satisfactory. In brief, a two blocks factorial design was given by the system at different factor levels. Formulations were made accordingly with the drug as 30 mg/10 g of drug and the data was plotted in each response (see Table 2). The program generated a quadratic equation as Y = b_0_ + b_1_X_1_ + b_2_X_2_ + b_3_X_1_X_2_ where b_0_, b_1_, b_2_, b_3_ and X_1_X_2_ are the intercept, linear-coefficients, and interaction term respectively, for the response Y (Table 3).

#### 2.1.3. Responses Evaluation

##### Vesicle Size (nm): Y_1_

**Figure 2:**
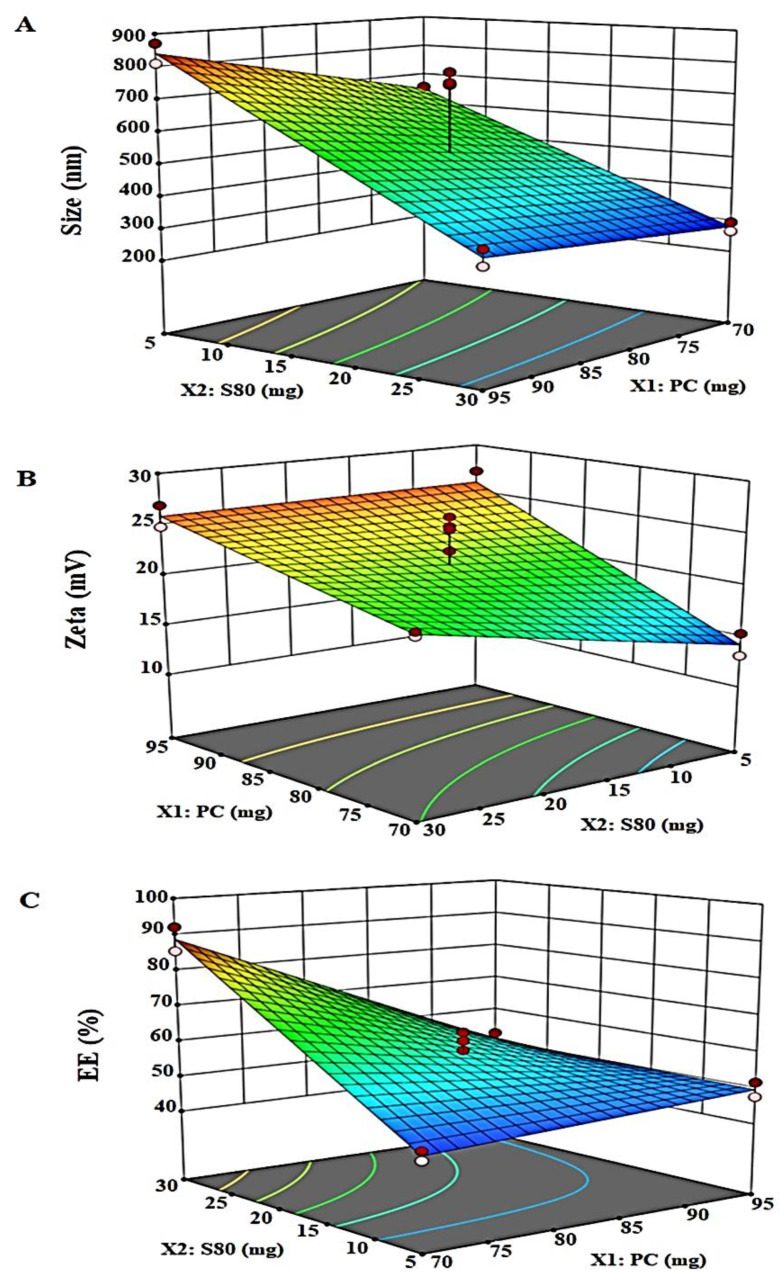
Design Experiment 3-dimensional response surface plots of the vesicle size (Y_1_), zeta potential (Y_2_), and % EE (Y_3_). (**A**) Three-dimensional response surface plot of (Y_1_) depicting minimal change in size with increase in PC content at high surfactant level. However, the size increases with decrease in Span 80 content at any PC levels. (**B**) Three-dimensional response surface plot of (Y_2_) depicting changes in zeta potential with change in PC and Span 80 levels. Here, the lowest value observed at low levels of both factors. (**C**) Three-dimensional response surface plot of (Y_3_) which revealed a significant increase in the % EE of LUT at lower level of PC and higher level of Span 80.

The size of any elastic liposomal formulation is a critical parameter for developing formulations for topic use. The approached is established and validated for various drugs. However, elastic liposomes (ELs) have unique characteristics over conventional liposomes such as deformability, flexibility of the vesicle membrane, adaptability to stress, and sensitivity to the water gradient of skin [23,24]. This uniqueness allows for better squeezing through smaller skin pores. The vesicle size values of the proposed twelve runs and the polynomial quadratic equation for the response Y_1_ are presented in Table 2 and Table 3. Each term has either positive or negative signs indicating either a synergistic or antagonistic effect of the response Y_1_. Particle size ranged between 265–871 nm and the quadratic equation was Y_1_ = 527.9 + 62.87X_1_ − 217.63X_2_ − 32.12 X_1_X_2_. Figure 2A illustrates the response surface plot of Y_1_. The quadratic equation was the best fit model based on an analysis of variance (ANOVA) report and showed *p* (0.0001), high value of F (204.2) and r^2^ value of 0.99 (Table 3). This mathematical model of the response (Y_1_) on factors (X_1_ and X_2_) showed that the vesicle size significantly (*p* < 0.05) increases with a decrease in Span 80 content at any content of PC. However, the highest vesicle size was observed at highest level of (X_1_) and lowest level of (X_2_). The value of the adjusted regression coefficient (r^2^) (0.99) was in agreement with the predicted (r^2^) (0.95), suggesting a good model fit. Therefore, Y1 can be optimized by increasing Span 80 content and lowering PC content (size at minimum ~ 265 nm).

##### Zeta Potential (ZP): Y_2_

The electric potential difference in vesicle double layer and media is called Zeta potential. Higher ZP values (nearly ± 30 mV) represent a good stability in colloidal system [25]. The values of PC and Span 80 based on the design of experiment (12 runs) formulations showed in the range of −13 to −27 mV. The quadratic equation was Y_2_ = 21.58 + 4.73X_1_ + 1.45X_2_ − 1.55 X_1_X_2_. Figure 2B shows the response surface plot of Y_2_. The quadratic model was the best fit and supported with ANOVA report [*p* (0.0006), high F (28.7) and r^2^ values (0.93) (Table 3)]. This mathematical model of the response (Y_2_) on factors (X_1_ and X_2_) showed that zeta potential significantly (*p* < 0.05) increases with increase in PC and Span 80 contents. The value of adjusted regression coefficient (r^2^) (0.90) and predicted (r^2^) (0.72) suggesting enough power for model fit. Here, values of zeta potential were found to be significantly higher with higher PC levels regardless of Span 80 content. However, the zeta potential decreased the most when both factors were at lower levels.

##### Percentage Entrapment Efficiency: Y_3_

**Figure 3:**
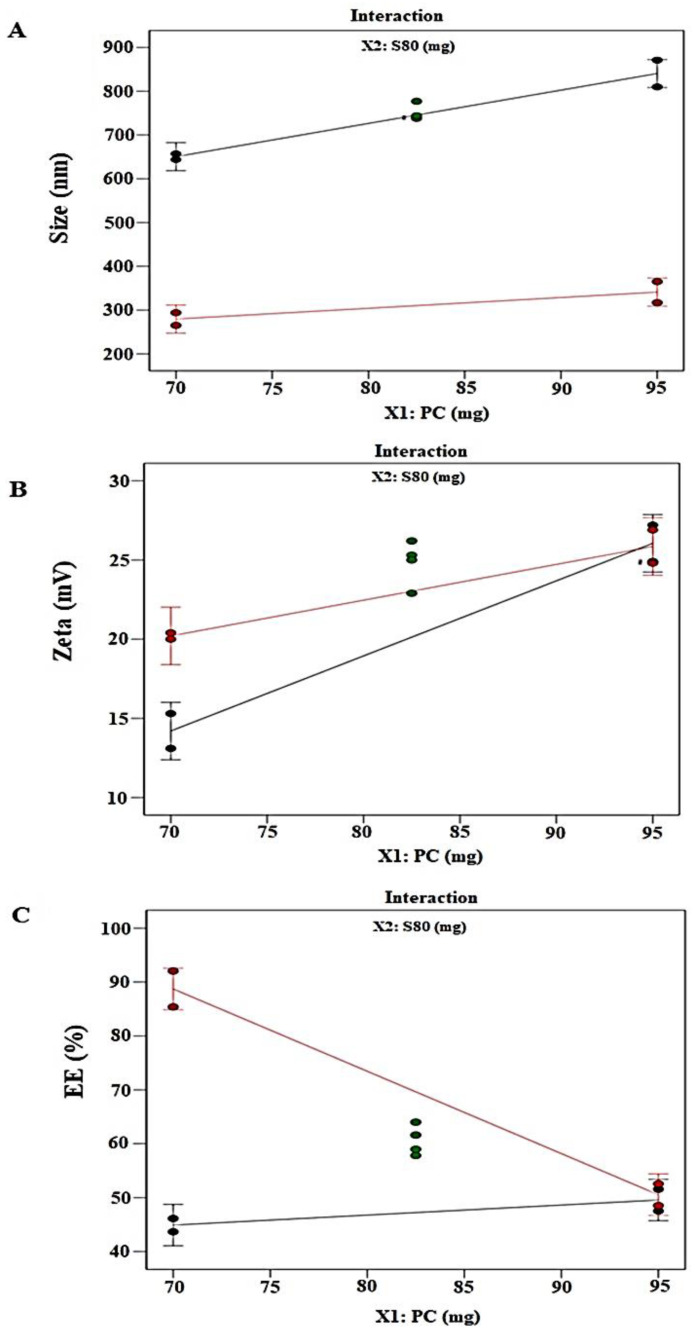
Design experiment factor interaction plots of (**a**) the vesicle size (Y_1_), (**b**) zeta potential (Y_2_), and (**c**) % EE (Y_3_). Green dots represent center points.

LUT is a hydrophobic drug with a log *p* of three. Thus, LUT is anticipated to be entrapped in the layer (lipid bilayer) ELs vesicles through lipophilic–lipophilic interaction. The percentage of entrapped LUT in the elastic liposomal formulation was found in the range of 43.7–92.1% (Table 2). Here, the %EE increased with an increase in Span 80 content which can be attributed to the flexibility that Span 80 added to the vesicles. Varshosaz et al. claimed that Span 80-containing niosomes exhibited a lower %EE of insulin, indicating that LUT will be entrapped at a higher percentage within the lipophilic lipid bilayers of vesicles [26]. Generally, lower HLB-based surfactant is appropriate for high % EE of hydrophobic drugs and vice versa. Thus, these effects are attributed to increased %EE of LUT in elastic liposomes containing Span 80. A software generated the mathematical quadratic equation of Y_3_ = 58.43 − 8.39X_1_ + 11.2X_2_ − 10.71 X_1_X_2_, which established a relationship between the response (%EE) and the independent variables (X_1_ and X_2_) (Table 3). This generated equation for Y_3_ is valid as evidenced by the statistical values of *p* (0.0001) and f (83.67). Figure 2C depicts the 3-D surface plot of Y_3_ against X_1_ and X_2_. ANOVA analysis report found a good agreement between the adjusted r^2^ (0.97) and predicted r^2^ (0.90) indicating a good fit of the quadratic model for Y_3_. Thus, at lower content of X_1_ and higher content of X_2_, the maximum %EE of LUT will be obtained. There were no significant interactions existing between the factors and responses as shown in Figure 3A–C.

#### 2.1.4. Desirability

**Figure 4:**
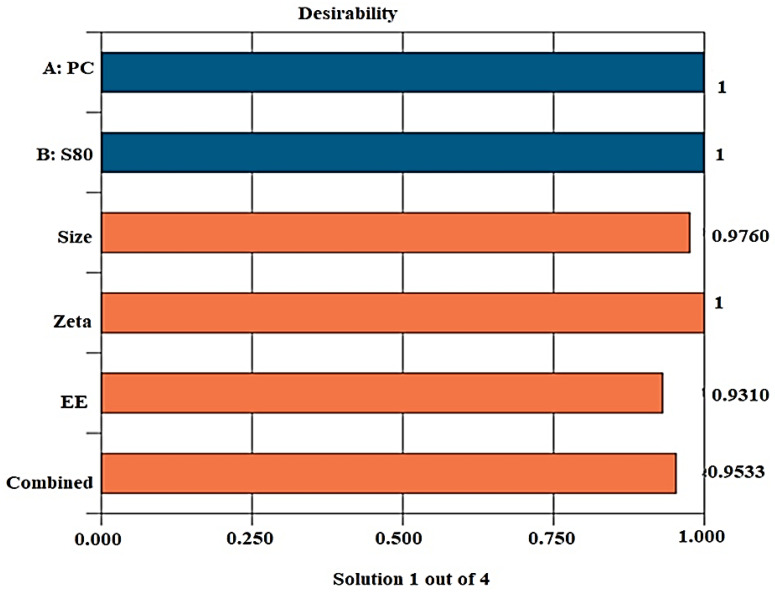
The desirability function of the optimized proposed formulations (OLEL1).

This numerical objective function is applied for validation of the optimization process. Here, the optimized formulations is identified under a specific set of constraints and importance is given to the independent and dependent variables [27]. This function suggested four formulations with specific levels of X_1_ and X_2_. Experimental values of vesicle size, PDI, and %EE for optimized formulation OLEL1 with desirability function values of 0.95 is observed as shown in Table 4. The observed values were found to be in agreement with predicted values suggesting the best fit of the model. The overall desirability approached an approximate value (0.95) of unity which indicated the suitability of the model for the optimization process (Figure 4).

#### 2.1.5. Morphological Assessment

**Figure 5:**
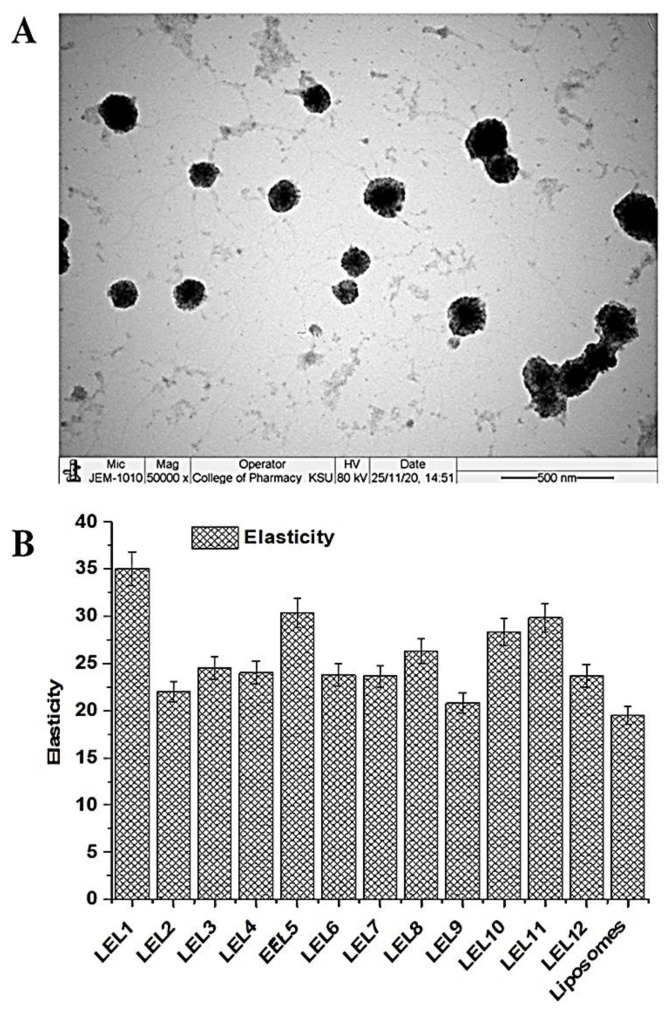
(**A**) Morphological study of OLEL1, using TEM, (**B**) elasticity of developed LUT-loaded elastic liposomes (LEL1-LEL12) and comparison against liposomes.

The optimized elastic liposome OLEL1 was visualized under TEM for morphological study (Figure 5A). Here, the vesicles appeared to be spherical in shape, apparently dispersed, and uniformly distributed in the colloidal dispersion of elastic liposomes. Also, this showed that the colloidal suspension of elastic liposomes was stable without sign of phase separation and aggregation. Moreover, there were no drug precipitation in the colloidal suspension. It is clear in the image that the outer lipid bilayer is firm and stable, composed of the explored lipid and surfactant.

#### 2.1.6. Elasticity

The proposed vesicular carrier system is devoid of cholesterol and expected to bear maximized ultra-deformability under stress conditions. Therefore, it is expected to have relatively high flexibility due to the combined effect of plasticizer (7% ethanol), and Span 80 (serving as edge activator). Cholesterol provides a stern and firm strength to the lipid bilayer of liposomes due to which it is considered as relatively more rigid compared with elastic liposomes [28]. The result of elasticity of all elastic liposomes and liposomes is portrayed in Figure 5B. Total twelve elastic liposomes loaded with LUT were prepared (LEL1–LEL12) as per suggested block (Table 2). All of the elastic liposome formulations exhibited significantly (*p* < 0.05) higher elasticity (in the range of 20.6 ± 1.0–35.5 ± 1.3) as compared with liposomes (*E* = 18.3 ± 0.7) (Figure 5B). There was a remarkable impact of Span 80 concentration relative to PC for elasticity. LEL1, LEL5, LEL10, and LEL11 exhibited higher elasticity among them which may be attributed to high content of Span 80 (30 mg) as compared with others. Likewise, LEL3, LEL6, LEL8, and LEL12 revealed relatively low elasticity as evidenced by a low content of Span 80 (5 mg). Formulations LEL2, LEL4, LEL7, and LEL9 exhibited elasticity in the range of 20–23.7. This may be attributed to cholesterol free vesicular lipid bilayer and ethanol mediated fluidity imparted to the layer. There are several factors controlling the elasticity of lipid vesicles such as composition, hydrocarbon chain of lipid, types of edge activator, polarity of head group of lipid and surfactant, glass transition temperature of lipid, and glycerol bridge as link of acyl hydrocarbon [29]. Moreover, molecular weight, degree of unsaturation in hydrocarbon, cholesterol content, transition temperature, and the nature of surfactant all have a collective influence in modulating the fluidity and flexibility of the lipid bilayer of the vesicle’s system for enhanced permeation across the microscopic pores of human skin [28].

#### 2.1.7. In Vitro Drug Release Study

**Figure 6:**
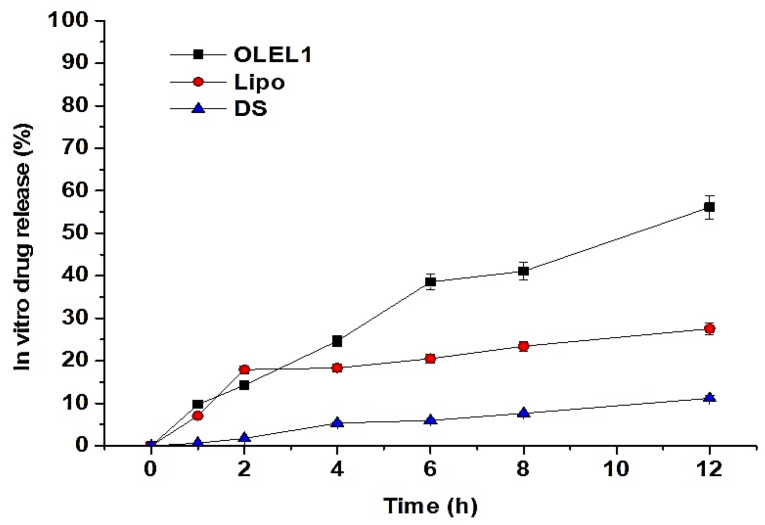
In vitro drug release pattern of the optimized elastic liposome formulations (OLEL1) as compared with conventional liposome (lipo) and drug solution (DS) over period of 12 h. OLEL1 and lipo showed significant difference (*p* < 0.05). Data presented are mean ± s.d (*n* = 2).

The percentage of LUT released over 12 h for OLEL1, lipo, and DS are depicted in Figure 6. OLEL1 exhibited maximum release over period of 12 h which was attributed to the optimum content of X_1_ (PC = 70 mg) and X_2_ (Span 80 = 30 mg). In the first two hours there were no significant differences between OLEL1 and lipo in LUT release. Moreover, OLEL1 exhibited a slow and sustained release over the experimental time period with a maximum released at 12 h of ~56%. However, both lipo and DS showed only ~27% and ~11% at 12 h, respectively. In a previous report, Abidin et al. claimed approximately 80% LUT release from control gel within 12 h which was due to ethanolic solution of LUT [14]. In the present study, DS exhibited a limited release of the drug over a period of 12 h which is due to the poor aqueous solubility of LUT at the studied temperature. However, improved release of the drug from the elastic liposome may be prudent to correlate with increased solubilization of LUT in the lipid bilayer of the vesicle, subsequently resulting in a slow and sustained release behavior. Controlled release may be attributed to the lipid bilayer serving as a rate limiting membrane. Comparing with liposomes, liposomes exhibited a 2.07-fold slower release than OLEL1 due to cholesterol-based rigid vesicle [28]. Flavonoid loaded liposomes are challenged with physical stability and drug leakage after long term storage. This stability depends upon the orientation of the flavonoid (LUT) in the lipid bilayer membrane of liposomes involving lipophilicity, and planar geometry [15]. These two properties resulted in a decreased permeability of the lipid bilayer membrane, a high affinity of LUT to liposomes, and a rigidifying role on the membrane [30,31].

#### 2.1.8. Ex Vivo Permeation and DD Studies across Rat Skin

**Figure 7:**
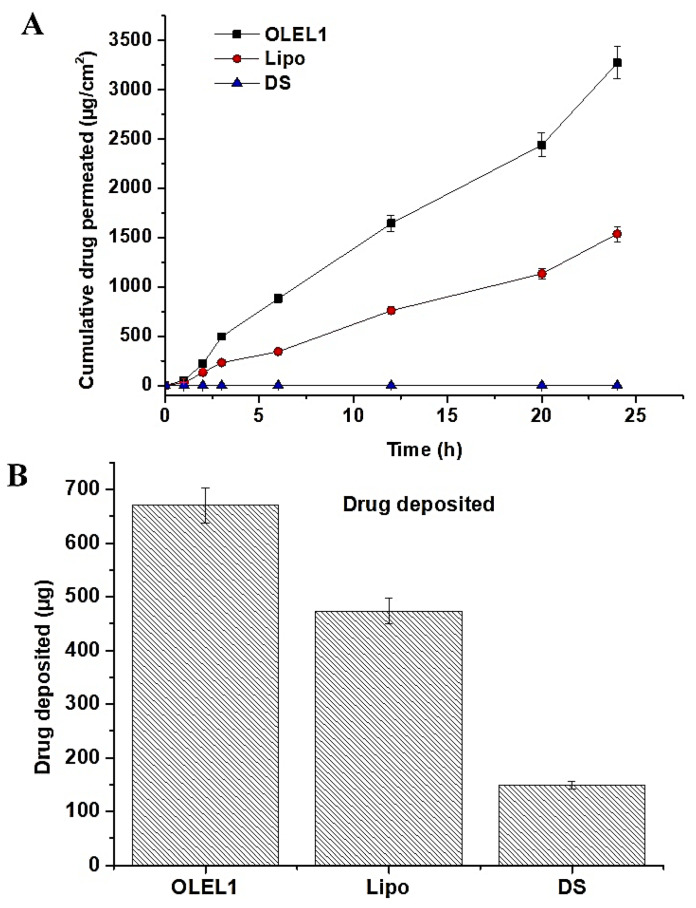
(**A**) Ex vivo LUT release pattern of the optimized elastic liposomes formulations (OLEL1) as compared with conventional liposome (lipo) and drug solution (DS) over a period of 24 h. Data presented are mean ±s.d (*n* = 2), and (**B**) drug deposition study of OLEL1, lipo and DS into the skin after 24 h of permeation study. Data presented are mean ± s.d (*n* = 2).

Permeation behavior of several drugs across human skin remained a challenging task due to the unique physiological feature of stratum corneum (SC) as a critical barrier [32]. In this study, an LUT vesicle-based approach was utilized for transdermal delivery using rat skin over 24 h (Figure 7A). Optimized formulations for OLEL1 and lipo showed permeation values of ~3270 µg/cm^2^ and ~1536 µg/cm^2^ across rat skin at 24 h, respectively. This demonstrated a 6.2- and 2.9-fold increase over DS. The OLEL1 release rate was expected to be controlled by the lipid bilayer as the controlling factor, with the SC layer as the main rate-controlling physiological factor [32]. This is associated with the hydrophobic nature of the drug and its likely compatibility with the hydrophobic SC layer of the skin. In addition, LUT-loaded OLEL1 exhibited significant permeation which may be due to the small size of the vesicles, high drug entrapment, and profound fluidity in elastic liposomes as compared with other liposomes. Span 80 is associated with the unsaturation in long chain hydrocarbons of oleate ester (presence of double bond) which causes a disturbance in the packing chain of the edge activator. This disturbed packing in the lipid bilayer results in increased fluidity, flexibility in the lipid bilayer, elasticity, and ease of squeezing across SC and microscopic pores [33]. This suggested that the encapsulated LUT was successfully permeated through the epidermis using mechanisms such as deformability, squeezing solubility in skin lipid, and hydration effect [23]. Furthermore, the rat skin permeation flux values of OLEL1, and lipo were found to be 136.3, 64 and 24.3 µg/h/cm^2^, respectively (Table 5). The calculated values of enhancement ratio for OLEL1 and lipo were 5.6 and 2.6, respectively. These results are in agreement with the published report of LUT-loaded niosomal gel wherein the enhancement ratio achieved was 2.66, similar to the liposome-based product in our case [14]. In contrast, the optimized OLEL1 achieved a 1.5 times higher permeation flux value as compared with the published niosomal LUT (93.21 µg/h/cm^2^) across skin [14]. The lag time was, also, significantly lower for OLEL1 (2 h) compared with lipo and DS, at 4.5 and 4, respectively.

Results of the drug deposition study are presented in Figure 7B. OLEL1, lipo and DS formulation deposited LUT as 670 µg (22.33%/cm^2^), 473 µg (15.76%/cm^2^) and 148 µg (4.9%/cm^2^), respectively. It is apparent that for maximum LUT deposition higher permeation flux is needed as found in OLEL1. Moreover, OLEL1 and lipo showed 4.5- and 3.2-fold increases in drug deposition as compared with DS. In general, the more the value of drug deposition, the more permeation flux is expected due to drug deposit formation in the dermal layer. Thus, the elastic, deformability, flexibility, and fluidity behaviors of the vesicle membrane, and permeation improvement through surfactant and plasticizer of elastic liposomes, supported drug deposition and subsequent permeation flux of LUT [34].

In our recent publication, we reported cation nanoemulsion for transdermal delivery of LUT using bergamot oil (as organic phase), cremophor-EL (surfactant), labrasol (as surfactant) and oleylamine as positive charge inducer [34]. On comparing cationic nanoemulsion (CNE4) with anionic nanoemulsion, the imposed cationic charge enhanced the transdermal permeation profile across rat skin. It is interesting that the elastic liposome-based formulation achieved the same permeation flux (136.3 µg/cm^2^ h) without this charge imposed on the vesicle surface or cationic lipid. However, the optimized elastic liposome “OLEL1” was found to have a higher drug deposition value (22.33%/cm^2^) as compared with the previously reported cationic CNE-4 (10.98%/cm^2^) [34]. Thus, the augmented flux and drug deposition of LUT may be attributed to the ultra-deformability and flexibility of elastic liposomes (free from cholesterol content) as compared with cholesterol based liposomes. In addition, it may be prudent to correlate the high drug deposition of OLEL1′s vesicular nature and high drug entrapment as compared with cationic nanoemulsion.

#### 2.1.9. Cytotoxicity Study

**Figure 8:**
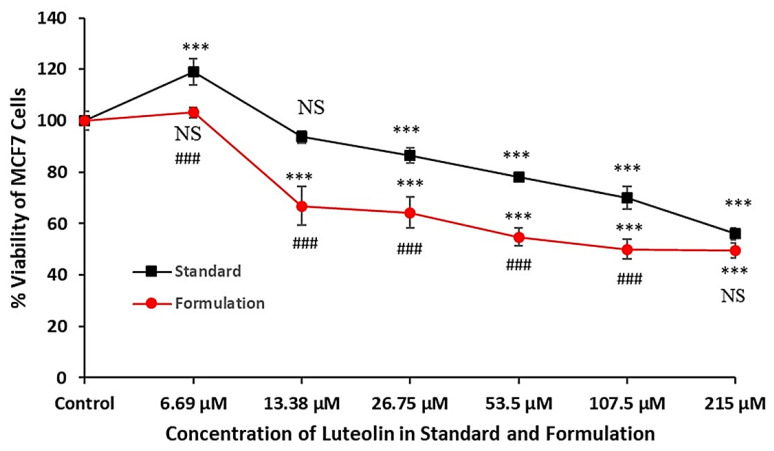
Effect of different concentrations of luteolin standard and luteolin formulation (OLEL1) on viability of MCF7 cells evaluated by MTT assay. Data are presented in percent (%) in comparison with control as 100%. Tukey test was utilized to analyze statistically significant difference between different concentration exposures and control. Difference was considered significant if *p* value was found to be <0.05. NS = not significant when compared with control; *** = *p* < 0.001 when compared with control; NS = not significant when compared with same concentration group of luteolin standard; ### = *p* < 0.001 when compared with same concentration group of luteolin standard.

Data reveal that both LUT standard and LUT formulation exhibit concentration dependent effects on the cell viability of MCF7. Cell viability (%) for different LUT standard concentrations was 118.95 ± 5.09 (6.69 µM,), 93.64 ± 2.37 (13.38 µM, *p* < 0.05), 86.4 ± 3.0 (26.75 µM, *p* < 0.005), 78.22 ± 0.52 (53.5 µM, *p* < 0.005), 69.94 ± 4.47 (107.5 µM, *p* < 0.005) and 56.0 ± 2.45 (215 µM, *p* < 0.005). Cell viability (%) for different concentrations of LUT formulation was 103.09 ± 1.9 (6.69 µM,), 66.81 ± 7.44 (13.38 µM, *p* < 0.05), 64.28 ± 5.91 (26.75 µM, *p* < 0.005), 54.81 ± 3.34 (53.5 µM, *p* < 0.005), 50.05 ± 3.91 (107.5 µM, *p* < 0.005) and 49.6 ± 2.91 (215 µM, *p* < 0.005). On comparing the same concentration groups in both, the LUT formulation exhibited significantly higher efficiency against MCF7 cell viability as compared with LUT standard (*p* < 0.001), except in the 215 µM concentration group. When comparing the effects, it clearly appears that the formulation of LUT has enhanced growth inhibitory effects in MCF7 cells (Figure 8). In the present investigation, the IC_50_ of the LUT standard in MCF7 cells was found to be 216.81 µM, which is reduced by the formulation to 164.4 µM that is 1.31 times lower than standard LUT, something which may be due to the short incubation time (4 h). MTT assay, or cell viability assay, revealed that the LUT has concentration dependent inhibitory effects on the growth of MCF7 cells. These effects indicate the cytotoxic nature of the LUT against cancer cells in vitro and can be exploited for further investigation. Data from the cell viability assay also highlighted that the LUT-containing formulation has significantly enhanced these effects in terms of reducing the IC_50_ as compared with standard LUT. The blank formulation did not show any cytotoxicity against MCF-7 cells which may be due to biocompatibility regarding the phospholipid and nonionic surfactant. In the present study, the cytotoxicity behavior of LUT was investigated for short incubation time (30 min). However, the formulation illustrated a rapid reduction in viable cells after treatment as compared with pure drugs. For further advancement in the current work, we need to investigate concentration- and incubation time-dependent cellular inhibition (antitumor potential) against the same cell lines. Jeon and Suh investigated the synergistic antiapoptotic effect of celecoxib and LUT on breast cancer cells followed by varied incubation time against the same cell lines [35].

## 3. Materials and Methods

### 3.1. Materials

Luteolin (LUT) was purchased from Beijing Mesochem Technology Co. Pvt. Ltd. (Beijing, China). Phospholipon^®^ 90G (P-90G) (GmbH, Nattermannallee 1, Koln, Germany) is chemically phosphatidylcholine (PC) containing ascorbyl palmitate (0.1%). Span 60, Span 80 and Brij 35 were procured from Thermo-Fisher Scientific (Waltam, MA, USA). DMSO (VWR Chemicals, France), MTT (3-(4,5-Dimethylthiazol-2-yl)-2,5-Diphenyltetrazolium Bromide) (Invitrogen, Thermo Fisher, USA), Advanced DMEM (Dulbecco’s Modified Eagle Medium) (Gibco, Life Technologies Ltd., London, UK), NaCl (sodium chloride), KCl (potassium chloride), Na_2_HPO_4_ (disodium hydrogen phosphate) and KH_2_PO_4_ (potassium dihydrogen phosphate) were procured from Scharlab S.L., Barcelona, Spain. Millipore water was used as an aqueous medium.

### 3.2. Preparation of Luteolin-Loaded Elastic Liposomes (LELs) Using Various Surfactants

The elastic liposomes (ELs) were formulated using a rotary evaporation technique (RET) [13]. PC and surfactants were first dissolved in a mixture of methanol-chloroform (1:2 ratio). Formulations were prepared using different surfactants namely Span 60 (F1, F2, F3), Span 80 (F4, F5, F6) and Brij 35 (F7, F8, F9) with different PC to surfactant ratios. The selected ratio 95:5, 85:15 and 70:30 were applied for each surfactant. Briefly, precisely weighed excipients and the drug (30 mg) was completely dissolved in a round bottom flask (RBF) containing methanol-chloroform mixture (1:2) (3 mL). The RBF went under evaporation of moderate temperature (40 ± 2 °C) and reduced pressure leading to a thin film (on inner surface). The film was hydrated with hydro alcoholic (0.7% *v*/*v* ethanol as plasticizer) PBS (10 mL, pH 5.5) solution. Thus, obtained colloidal milky elastic liposome formulations were sonicated (60 s) to reduce vesicle size [29,36,37]. Eventually, these were preserved in a freezer to activate vesicles (12 h). Each g of formulation contains 3 mg of LUT (0.3% *w*/*w*).

### 3.3. Vesicle Size and Size Distribution (Polydispersity Index, PDI)

Each formulation was individually assessed for size and PDI using a Zetasizer Nano ZS (Malvern Instruments, Worcestershire, UK) equipped with 4.0 mW He Ne red laser (633 nm) [37]. The samples were previously diluted (100 times) using water (milli-Q) to avoid instrument al error during analysis. The experiment was performed at 25 ± 1 °C and scattering angle of 90°.

### 3.4. Experimental Design Tool (Design Expert^®^)

Finding the optimum content of excipients in a formulation is called optimization. Therefore, Design-Expert 13.0.5 software was used to design the experiment (Table 2). Two blocks factorial design with four center points (12 runs) was employed to explore the design space for the selected factors and responses. This would later allow the software to predict the optimized formulation(s). This “Design Expert software” follows a random order for desired combination(s) to maximize the chance of identifying variation between runs. In this study, PC (X_1_) and Span 80 (X_2_) were selected as independent factors (variables) against four dependent variables. These responses were vesicle size (Y_1_), Zeta potential (Y_2_), and %EE (Y_3_). Both dependent and independent variables values are shown in Table 2. Smaller size liposomal formulations have a better chance to pass through the microscopic pores of the skin due to high elasticity. However, an optimum concentration of excipient is opted for safety concern and maximized delivery of LUT (0.05% *w*/*w*). Hence, two levels of PC were selected as 70 mg (low) and 95 mg (high) whereas Span 80 was set at 5 mg (low) and 30 mg (high).

In addition, the regression equation showing the best fit using the selected mathematical models was used and validated by equating various statistical parameters such as *p* value, regular, adjusted and predicted correlation coefficient (r^2^) [38,39]. Polynomial equations generated 3-dimensional surface and contour graphs were produced by the software. *p* and F values were used to assess model suitability while the optimization process was assessed using individual (*d_i_*) and overall desirability function (D_i_). Table 3 summarizes details of independent (X_1_ and X_2_) and dependent variables (Y_1_ to Y_3_). This value depends on several independent variables and set goals. 

### 3.5. Formulations Characterizations

#### 3.5.1. Vesicle Size and Zeta Potential

All prepared formulations were prepared and evaluated for size, polydispersity index (PDI) and surface charge (zeta potential, mV). Vesicle size and size distribution were assessed as per method reported in previous section. All of the formulations were assessed for zeta potential as an essential parameter controlling stability of the product. The sample was diluted (100 times) with water to disperse vesicles in the medium before size and PDI determination. In case of zeta analysis, the liposomal colloid was processed as such without dilution.

#### 3.5.2. Percentage Entrapment Efficiency (% EE)

This was determined by the reported procedure [40,41]. Developed formulations were placed aside overnight at 4 °C. Un-entrapped (free drug) was determined using Eppendorf tube (2 mL) centrifugation method (15,000 rpm for 15 min). The procedure was repeated to remove free drug completely. LUT contents were determined using a HPLC technique at λ_max_ of 350 nm. Finally, the entrapped drug in the vesicles was calculated by Equation (1):

% EE = [(Q_t_ − Q_s_)/Q_t_)] × 100 (1)

where, “Q_t_” and “Q_s_” were the added theoretical content of the drug added and the content of LUT present in the clear supernatant, respectively.

#### 3.5.3. Desirability Function Parameter and Validation

Desirability is a numerical function with the objective to reach the value of 1 leading to the best fit within expected constraints and goals. This is, also, used to identify interaction between factors if it exists. Statistically, “Di” is a geometrical mean function of the explored responses depending upon set “maximum”, “minimum”, “in range” “equal to”, and “target” ranges by the investigator during optimization process (Equation (2)):

D_i_ = (*d_*1*_. d_*2*_. … d_n_*) = (ᴨ_ii = 1_ *d_i_*)^1/n^ (2)

#### 3.5.4. Morphological Assessment

The formulation with highest desirability value (OLEL1) was visualized under transmission electron microscopy (TEM) (JOEL JEM1010, Japan). The sample (2–3 drops) was kept on a glass coverslip and dried overnight. Then, the same sample was kept on the carbon-coated copper grid for complete drying. Osmium was used to stain the lipid components and left for 24 h to dry. Then, the sample was loaded into the TEM to be viewed under different magnifications.

#### 3.5.5. Elasticity

Elasticity was measured following the reported method [42,43,44]. Briefly, elastic liposomes (LEL1–LEL12) and liposomes (as control) were extruded through a 50 nm pore-sized membrane (r_p_) for 10 min under 2.5 bar pressure. The extruded volume (J) and the mean diameter of the vesicles after extrusion (r_v_) were determined. Thus, the elasticity (E) of vesicles was calculated using Equation (3):

E = *J* × (r_v_/r_p_)^2^ (3)

#### 3.5.6. In Vitro Drug Release (%DR)

OLEL1, control liposome (lipo) and drug suspension (DS) prepared using 0.1% *w*/*v* sodium CMC (Na-carboxymethyl cellulose), were studied to understand their %DR profile. The study was conducted using a dialysis membrane (molecular cut-off 12–14 KDa, Himedia Labs). Each formulation and control samples (2 mL containing 6 mg LUT) were separately placed in the membrane tied from each ends using clip. The sample containing membrane bag was suspended in a beaker previously filled with 400 mL of PBS (pH 7.4) set at 37 ± 1 °C and constant stirring (100 rpm) using magnetic bead. The sample for analysis (3 mL) was withdrawn at 1, 2, 4, 6, 8, and 12 h to estimate the drug concentration released in the medium using a U.V. spectrophotometer at 350 nm.

### 3.6. Analytical Method

The quantitative assessment of LUT was performed using a validated high performance liquid chromatography (HPLC) technique [45]. In this, the packing material of the analytical column (150 mm × 4.5 mm) worked as stationary phase with particle size of 5 µm operating at 30 ± 1 °C. The sample was injected at low volume (20 µL) for 8 min (run time) at flow rate of 1 mL/min. For quantitative assessment, the mobile phase (MP) was freshly prepared using acetonitrile, methyl alcohol, and aqueous (including 1%*v*/*v* acetic acid). These components were prepared in 60:30:10% *v*/*v,* ratio. The prepared MP was set at pH 4.0 and subsequently passed through a membrane filter to retain any fibers and particles (if found). The drug analysis was conducted on an isocratic mode using a UV detector (350 nm as set wavelength). A working calibration curve was constructed over concentration range of 20–100 µg/mL with high regression coefficient (r^2^ > 0.99) [45].

### 3.7. Ex Vivo Drug Permeation and Deposition Study

This study was carried out using rat skin (excised from abdominal portion) (body weight of ~200 g albino male rats) from the Animal Center, College of Pharmacy, King Saud University, Riyadh, Saudi Arabia. Approval (2 December 2020) was issued from the Institute’s Ethics Committee (King Saud University, Riyadh) (KSU-SE-20-64). This experiment was conducted based on the guideline for animal care (NC3Rs, ARRIVE guidelines). Stratum corneum (SC) of rat skin has similar thickness to human skin and shows similarity in the permeation in different studies [46]. Thus, transdermal permeation of the optimized formulations (OLEL1), control liposome (lipo) and drug solution (DS) was conducted using a Franz diffusion cell. The collected skin was cleaned (free from hairs, and fatty matters) using an electric shaver. The skin was placed between both chambers where dermal side faced the receptor PBS medium (pH 7.4) and donor received the sample (LUT = 15 mg). The receptor medium was under regular stirring (rice bead, 100 rpm) and temperature of 37 ± 1 °C. Furthermore, sampling was carried out at 1, 2, 3, 6, 12, 20 and 24 h and estimated using HPLC (absorbance wavelength as 350 nm). Permeation flux, cumulative permeation, and drug deposition (DD) were calculated [28,47,48]. DD values were obtained after completion of ex vivo permeation (24 h). For this, the remaining sample on the surface was washed with running water and then sliced into small pieces. The drug was extracted using methanol: chloroform (1:2) mixture under constant stirring for 4h. The tissue was filtered and the filtrate was analyzed for the drug content [49,50,51,52].

### 3.8. Cytotoxicity Study Using MCF-7 Cell Lines

Effect on MCF7 cell viability of different concentrations of LUT standard and formulation was estimated by the MTT (3-(4,5-dimethylthiazol-2-yl)-2,5-diphenyl tetrazolium bromide). Then, MCF7 cell-lines (15,000 cells per well), were transferred to seed into the plates (96-well-oplates) along with 100 µL of the culture media (DMEM, 10% FBS). The same plates were required to incubate for overnight at 37 ± 1 °C and constant supply of 5% CO_2_ for complete adherence. To expose the cells to the compound, varied contents of LUT standard and the optimized formulation were poured into the respective wells. Standard LUT solution and formulation were prepared in DMSO (1%) and serially diluted using serum free media in a 96-well plate. The same concentration of DMSO was used in the vehicle control to avoid cell damage. The selected the drug concentrations in standard and formulation, were 215 µM, 107.5 µM, 53.5 µM, 26.75 µM, 13.38 µM and 6.69 µM. Cells were exposed to the samples (24 h), and then MTT solution (10 µL, 5 mg/mL PBS) was transferred to the respective wells. Furthermore, the culture was again incubated (4 h) so that the viable cells can metabolize MTT. The culture media was removed from each well followed by addition of DMSO solution (100 µL) to solubilize the formazan of MTT. Each plate was subjected for reading after 30 min of incubation at 570 nm. The DMSO solution was used as the blank.

### 3.9. Statistical Analysis

Experiments were performed in triplicate to get mean and standard deviation. All data were processed using Origin-pro (OriginLab Corporation, Northampton, MA, USA), and GraphPad version 5.01 software (La Jolla, CA, USA). Data were processed statistically using ANOVA (analysis of variance), Student’s *t*-test and a Turkey or Dunnett tests (Sigma Stat Software, 2.03, San Jose, CA, USA). The value was considered significant at *p* value of ≤0.05 for fitting the applied model followed by the correlation factor (r^2^).

## 4. Conclusions

The drug is challenging for formulation scientists due to low aqueous solubility, and poor oral bioavailability. Despite these limitations, the drug is well reported to have multiple therapeutic benefits, including for breast cancer (as discussed in the introduction section). Several scientists have applied techniques and carriers for improved efficacy of the drug to control breast cancer. However, no one has reported the use of vesicular elastic liposomes for transdermal delivery of LUT with improved efficacy against MCF-7 cell lines. The present study developed and optimized elastic liposomes and evaluated them for in vitro and ex vivo parameters. Results show that Span 80 was the most suitable edge activator amongst investigated surfactants (Span 60, and Brij) which may be due to negative glass transition temperature. Considering this, several formulations were dictated in Design Expert and characterized for vesicle size, zeta potential and %EE. Interestingly, these results were close to the predicted values suggesting the suitability of the model adopted for optimization (desirability ~ 0.95). TEM corroborated quite dispersed vesicles in colloidal system. Elastic liposomes showed Span 80 mediated elasticity. The optimized formulation illustrated facilitated drug release as compared with DS and rigid liposome. The proposed deformable vesicular carrier enhanced permeation parameters across rat skin as compared with DS and liposomes. Finally, the drug loaded carrier (OLEL1) exhibited concentration-dependent MCF-7 cells inhibition and elastic liposome improved cellular internalization for maximized inhibition as compared with control pure drug solution. Thus, the elastic liposomes can be a promising approach for improved transdermal delivery of LUT and enhanced therapeutic efficacy to control breast cancer.

## Figures and Tables

**Table 1:** A summary of preliminary formulations of elastic liposomes (F1–F9) using various types of surfactant and their characterization parameters.

Code	PC:S (% *w*/*w*)	Surfactant	HLB	T_g_ (°C)	Vesicle Size (nm)	PDI
**F1**	95:5 *	Span 60	4.7	53	358 ± 16	0.62 ± 0.05
**F2**	85:15	Span 60	4.7	53	284 ± 13	0.44 ± 0.03
**F3**	70:30	Span 60	4.7	53	187 ± 11	0.43 ± 0.02
**F4**	95:5 *	Span 80	4.3	−12	218 ± 9	0.45 ± 0.03
**F5**	85:15	Span 80	4.3	−12	212 ± 9	0.30 ± 0.01
**F6**	70:30	Span 80	4.3	−12	170 ± 6	0.35 ± 0.02
**F7**	95:5 *	Brij 35	16.9	40–45	385 ± 8	0.42 ± 0.03
**F8**	85:15	Brij 35	16.9	40–45	266 ± 5	0.35 ± 0.02
**F9**	70:30	Brij 35	16.9	40–45	234 ± 6	0.45 ± 0.04

Value represented as mean ± SD (*n* = 3), PC: S = phosphatidylcholine to surfactant ratio, HLB = hydrophilic lipophilic balance, T_g_ = glass transition temperature. * The minimum concentration where surfactant can form micelles. Values are reported at 25 °C. PDI: Polydispersity index

**Table 2:** Independent variables (X), responses (Y) and statistical models for luteolin-loaded elastic liposomes in experimental design.

Std	Block	Run	Factor 1 X_1_:PC (mg)	Factor 2 X_2_:Span 80 (mg)	Response 1 Size (nm)	Response 2 Zeta (mV)	Response 3 EE (%)
5	Block 1	1	70	30	265	20.4	85.39
9	Block 1	2	82.5	17.5	739	25.3	57.82
1	Block 1	3	70	5	644	13.1	43.67
10	Block 1	4	82.5	17.5	738	22.9	64
7	Block 1	5	95	30	317	26.9	52.55
3	Block 1	6	95	5	871	27.2	47.53
11	Block 2	7	82.5	17.5	777	26.2	58.97
2	Block 2	8	70	5	657	15.3	46.13
12	Block 2	9	82.5	17.5	744	25	61.61
8	Block 2	10	95	30	365	24.8	48.52
6	Block 2	11	70	30	294	20	92.06
4	Block 2	12	95	5	810	24.9	51.57

**Table 3:** Factors and responses and statistical parameters for luteolin-loaded elastic liposomes in experimental design.

	Experimental Design and Summary Reports		
Factors	Range	Goal	
X_1_: PC (mg)	70–95	In range	
X_2_: Span 80 (mg)	5–30	In range	
**Responses**			
Y_1_ (nm) as size	265–871	Minimum	
Y_2_ (mV) as zeta potential	−13.1––27.2	Maximum	
Y_3_ (%) as % EE	43.7–92.1	Maximum	
**Regression equations with best fitted model**			
Y_1_ = 527.9 + 62.87X_1_ − 217.63X_2_ − 32.12 X_1_X_2_			
Y_2_ = 21.58 + 4.73X_1_ + 1.45X_2_ − 1.55 X_1_X_2_			
Y_3_ = 58.43 – 8.39X_1_ + 11.2X_2_ − 10.71 X_1_X_2_			
**Statistical parameters**	**Y1**	**Y2**	**Y3**
r^2^	0.99	0.93	0.98
Adjusted r^2^	0.99	0.90	0.97
Predicted r^2^	0.95	0.72	0.90
Model f value	204.16	28.69	83.67
*p* value	0.0001	0.0006	0.0001
Model	Quadratic	Quadratic	Quadratic
SD	26.15	1.48	3.14
Mean value	601.75	22.67	59.15
% CV	4.35	6.54	5.32

Value of regression coefficient represented as r^2^, SD = Standard deviation, % CV = Coefficient of variation.

**Table 4:** Values of the predicted optimized and experimental formulations.

	PC	Span 80	Size	Zeta	EE%
Predicted	70	30	276	20.2	88.73
Experimental OLEL1	70	30	202	22.2	92 ± 3.8

**Table 5:** Ex vivo permeation parameters of luteolin-loaded formulations after 24 h.

Formulations	Jss^1^ (µg/cm^2^ h)	T_L_ (mean ± sd) (min)	Pc (mean ± sd) (cm/h)	ER^1^
OLEL1	136.26 ± 7.76	2.0 ± 0.01	1.36 × 10^−2^	5.61
Lipo	64.01 ± 0.91	4.5 ± 0.03	6.4 × 10^−3^	2.63
DS	24.31 ± 3.38	4.0 ± 0.02	2.3 × 10^−3^	-

Value represented as mean ± SD (*n* = 3). Jss^1^ = transdermal flux, calculated from the slope of Cartesian plot of cumulative amount of drug present in receptor compartment versus time. T_L_ = lag time (h). P_C_ = permeation coefficient = flux/the initial concentration of rifampicin dose applied to donor compartment. ER^1^ = enhancement ratio; This is the ratio of transdermal flux from the formulation to drug solution (injection solution).

## Data Availability

Not applicable.
